# Supplementary figures and images for: Long term cost-effectiveness analysis of IDegLira in the treatment of type 2 diabetes patients compared to GLP-1RA added to basal insulin after IDegLira entered the national reimbursement drug list in China
Source: PLoS One. 2025 Feb 6;20(2):e0310497. doi: 10.1371/journal.pone.0310497 (PMC11801598; doi:10.1371/journal.pone.0310497)

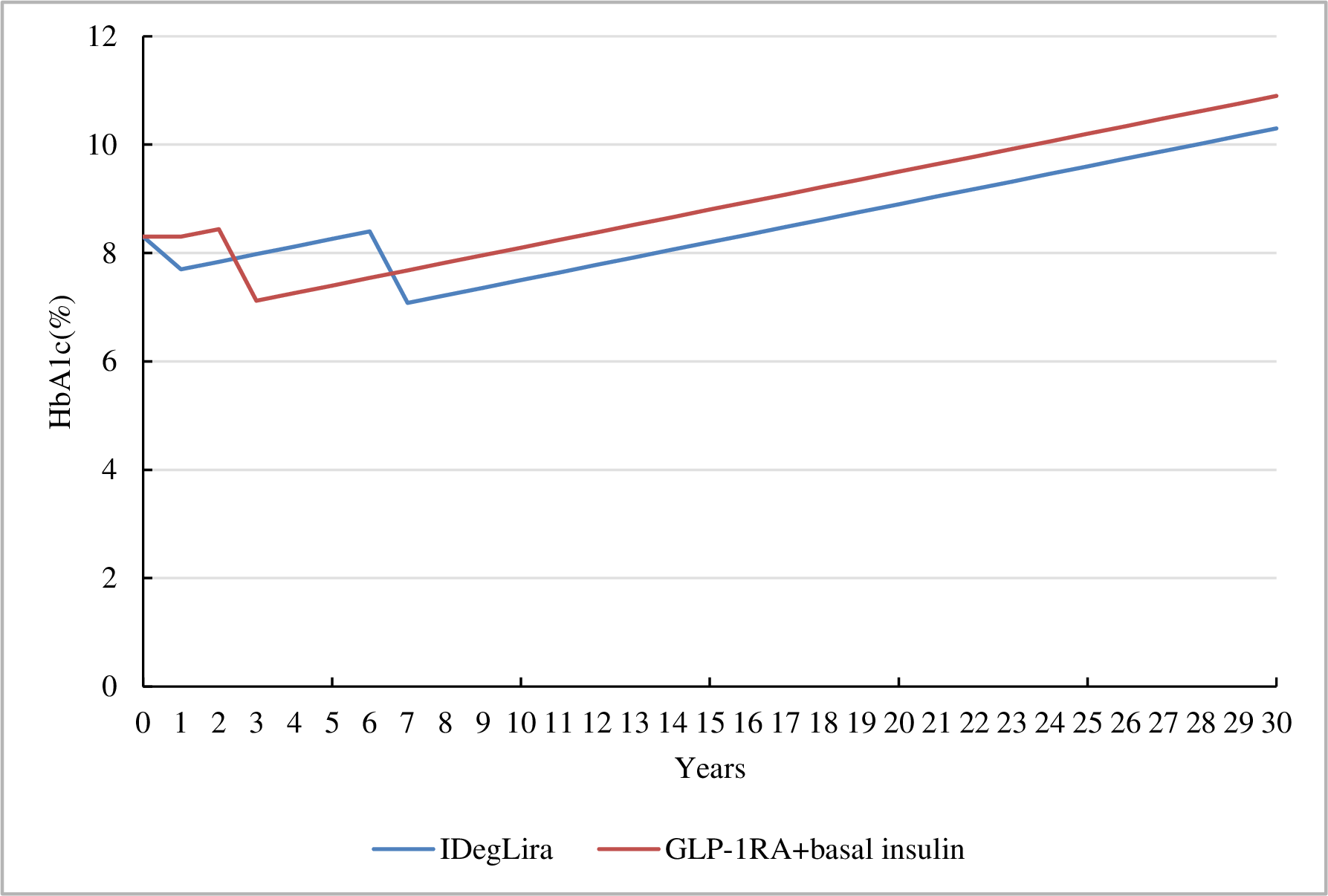

Supplement: S1 Fig — (TIF) [file pone.0310497.s001.tif]
