# Supplementary material for: Long term cost-effectiveness analysis of IDegLira in the treatment of type 2 diabetes patients compared to GLP-1RA added to basal insulin after IDegLira entered the national reimbursement drug list in China
Source: PLoS One. 2025 Feb 6;20(2):e0310497. doi: 10.1371/journal.pone.0310497 (PMC11801598; doi:10.1371/journal.pone.0310497)
Supplement: S1 Table — (DOCX) [file pone.0310497.s002.docx]

S1 Table. GLP-1RA cost

| N | GLP-1RA | Price (CNY) | Specifications | Dosage | Unit | Daily cost |
| --- | --- | --- | --- | --- | --- | --- |
| 10 | Exenatide | 408.00 | 10mcg | 17 | mcg | 11.56 |
| 6 | Exenatide once-weekly  formulation | 260.00 | 2mg*4*2 | 1.8/week | mg | 33.43 |
| 1 | Lixisenatide | 268.00 | 3ml；  Single injection20mcg(0.2ml) | 20 | mcg | 17.87 |
| 16 | Dulaglutide | 123.35 | 1.5mg | 1.5/week | mg | 17.62 |
| 108 | Liraglutide | 315.27 | 3ml:18mg | 1.6 | mg | 28.02 |
| Weighted average daily cost | | | | | | 25.8 |
