# Supplementary material for: Long term cost-effectiveness analysis of IDegLira in the treatment of type 2 diabetes patients compared to GLP-1RA added to basal insulin after IDegLira entered the national reimbursement drug list in China
Source: PLoS One. 2025 Feb 6;20(2):e0310497. doi: 10.1371/journal.pone.0310497 (PMC11801598; doi:10.1371/journal.pone.0310497)
Supplement: S2 Table — (DOCX) [file pone.0310497.s003.docx]

**S2 Table. Basal insulins cost**

| Basal insulins | Price after centralized procurement | Unit cost (CNY/U) |
| --- | --- | --- |
| Insulin degludec | 82.20 | 0.27 |
| Insulin detemir | 76.07 | 0.25 |
| Regular insulin U100 | 69.33 | 0.23 |
| Regular insulin U300 | 99.60 | 0.22 |
| Average cost | 81.06 | 0.24 |

Note: The daily dosage is 33.4U, and the daily basal insulins is 0.24*33.4=8.2 CNY
